# Effectiveness of pleurodesis for postoperative air leaks after lung resection

**DOI:** 10.1186/s13019-023-02444-6

**Published:** 2024-01-02

**Authors:** Norifumi Tsubokawa, Takahiro Mimae, Risa Ito, Ryuma Sasai, Kouichi Hirano, Atsushi Kamigaichi, Nobutaka Kawamoto, Yoshihiro Miyata, Morihito Okada

**Affiliations:** Department of Surgical Oncology, Hiroshima University, 1-2-3, Kasumi, Minami-ku, Hiroshima, 734-8551 Japan

**Keywords:** Lung cancer, Postoperative air leakage, Pleurodesis

## Abstract

**Background:**

Pleurodesis is often performed for air leaks; however, the ideal materials and timing of the procedure remain controversial. We investigated the efficacy of pleurodesis using different materials and timing.

**Methods:**

We retrospectively reviewed 913 consecutive patients who underwent segmentectomy or lobectomy for non-small cell lung cancer between 2014 and 2021. Pleurodesis efficacy was assessed on the day of chest tube removal.

**Results:**

Eighty-six patients (9%) underwent pleurodesis for postoperative air leaks. Pleurodesis was performed on a median of postoperative day (POD) 5. Talc was the most frequently used material (n = 52, 60%), followed by autologous blood patches (n = 20, 23%), OK-432 (n = 12, 14%), and others (n = 2, 2%). No difference existed in the number of days from initial pleurodesis to chest tube removal among the three groups (talc, 3 days; autologous blood patch, 3 days; OK-432, 2 days; P = 0.55). No difference in patient background, except for sex, was observed between patients who underwent pleurodesis within 4 PODs and those who underwent pleurodesis on POD 5 or later. Drainage time was significantly shorter in patients who underwent pleurodesis within 4 PODs (median, 7 vs. 9 days; P = 0.004).

**Conclusions:**

The efficacies of autologous blood patch, talc, and OK-432 would be considered comparable and early postoperative pleurodesis could shorten drainage time. Prospective studies are required.

## Background

Postoperative air leakage after lung resection is a common complication that may increase the length of hospital stay or occurrence of morbidity, such as empyema and pneumonia [1, 2]. Although it is a common complication, no standard treatment has been established for air leakage. Treatments for air leaks include drainage, chemical pleurodesis, autologous blood patches, surgery, and endoscopic interventions [3]. Pleurodesis is often performed for persistent air leaks that last longer than postoperative day (POD) 5–6 [4–6], despite lack of evidence for this timing.

Autologous blood patches are widely used; however, evidence for this has not been established. Two randomized trials [4, 5] revealed that autologous blood patches were more effective in sealing air leaks after lung resection than drainage; however, a recent randomized trial did not indicate any benefits for blood patch pleurodesis [6]. Although chemical pleurodesis, such as talc and OK-432, is reportedly an effective treatment for postoperative air leaks [7, 8], no prospective studies have been conducted. In addition, because no consensus exists on the adequate time to classify postoperative air leaks and the defined amount of air leaks that pleurodesis should be performed [3, 8, 9], it is unclear whether pleurodesis on POD 5 or later is appropriate. Pleurodesis is sometimes performed early in anticipation of sealing air leaks or delayed due to associated complications, including lung injury and acute respiratory distress syndrome [10, 11]. Therefore, the present study aimed to evaluate the efficacy of materials used for pleurodesis—autologous blood patch, talc, and OK-432—and investigate whether early postoperative pleurodesis (within 4 PODs) is more effective than standard pleurodesis (on POD 5 or later) in stopping air leaks after anatomical lung resection for non-small cell lung cancer.

## Methods

### Study cohort

Between January 2014 and December 2021, 913 patients who underwent complete anatomical resection for non-small cell lung cancer at Hiroshima University Hospital were retrospectively reviewed. Patients with postoperative air leakage on POD 2 or later were included in the study.

### Surgical procedure

Hybrid video-assisted thoracotomy (VATS) was mainly performed via a 6-cm muscle-sparing thoracotomy without a rib retractor. Open thoracotomy and robot-assisted thoracoscopic surgery were sometimes performed depending on the degree of adhesion and tumor status. The pulmonary hilum was exposed to visualize the branches of the pulmonary artery and vein. The vessels were dissected using an energy device or stapler. For lobectomy, the bronchus was secured to the lobe and resected. For segmentectomy, the area from the bronchus to the segments to be resected was secured, and an inflation-deflation line was used to delineate the intersegmental plane. After dissecting the bronchi, the lung parenchyma was dissected using electrocautery or staplers. Staplers were used in patients with interstitial pneumonia or emphysema. After lung resection and lymph node dissection, an intraoperative air leak test (20 cm H_2_O applied pressure) was performed by an anesthesiologist by expanding the remaining lung. The amount of intraoperative air leakage was assessed. When an air leak was detected, additional suture repair was performed. Additionally, fibulin glue, polyglycolic acid sheets, and/or collagen patches coated with fibrinogen and thrombin were used. A chest tube was then placed at the apex of the thoracic cavity. After surgery, we managed the chest tube under a water seal in a conventional drainage system and − 8cmH2O in a digital drainage system. All patients who underwent surgery before 2014 received a conventional drainage system, however, many patients from 2015 received a digital drainage system. The surgical approach and procedures were determined at a surgical conference based on the tumor and patient status.

### Pleurodesis

Sterilized controlled talc (Nobelpharma, Tokyo, Japan), a killed streptococcal preparation of OK-432 (Chugai Pharmaceutical, Tokyo, Japan), and autologous blood patches were the three materials mainly used for pleurodesis at our institution. Although pleurodesis is generally considered for an air leak lasting for more than 5 PODs, each surgeon decided on the timing of pleurodesis and on the material to use based on the patient’s status or the amount of air leaks. The patients with severe emphysema or interstitial pneumonia were often treated with autologous blood patch. In addition, in the early period of this study, pleurodesis was often performed with OK432 or talk. However, in the later period, autologous blood patch was used for pleurodesis. For autologous blood patch, 100 mL of peripheral venous autologous blood patch was directly injected into the pleural cavity through the chest tube, followed by saline flush solution. For chemical pleurodesis, 20 mL of 1% lidocaine hydrochloride (200 mg) was instilled into the pleural cavity through the chest tube, followed by a solution of 50 mL normal saline containing 4 g of sterilized controlled talc or 5 − 10 KE (Klinische Einheit) of OK-432 (where 1 KE contains 0.1 mg of dried cocci). The chest tube was raised about 50 cm above the patient to trap the agent but allow air to pass through the chest tube. Patients were repositioned every 15 min so that the agent could contact all pleural surfaces. The chest tube was lowered 2 h later. If patients needed painkillers, we prescribed Non-Steroidal Anti-Inflammatory Drugs or acetaminophen. If initial pleurodesis failed, we often administer the same materials on the 2nd pleurodesis. In addition, when multiple pleurodeses failed, surgery for pleurodesis was considered based on the patient status. When evaluating the efficacy of the different materials, we included only the first material if a patient received more than one. Since a prolonged air leak was defined as an air leak lasting for more than 5 PODs [12], early postoperative pleurodesis was defined as pleurodesis performed within 4 POD (early group), while standard postoperative pleurodesis was defined as pleurodesis performed after POD 5 or later (standard group) in this study. The efficacy of pleurodesis was assessed on the chest tube removal day, and differences among the three initial materials and between the early and standard groups were examined. Postoperative complications were defined as those occurring within 30 days after surgery with a Clavien–Dindo grade ≥ 3 [13].

### Statistical analysis

Continuous variables are reported as median (interquartile range [IQR]) and were compared using the Wilcoxon rank-sum test. Categorical variables are reported as numbers (percentages) and were compared using the chi-squared test. Logistic regression model was used to identify risk factor of requiring two or more pleurodesis. A backward stepwise method was used to select viables for multivariable analysis. Statistical significance was set at P < 0.05. All data were statistically analyzed using JMP software (version 16.0; SAS Institute, Cary, NC, USA).

## Results

**Fig. 1:**
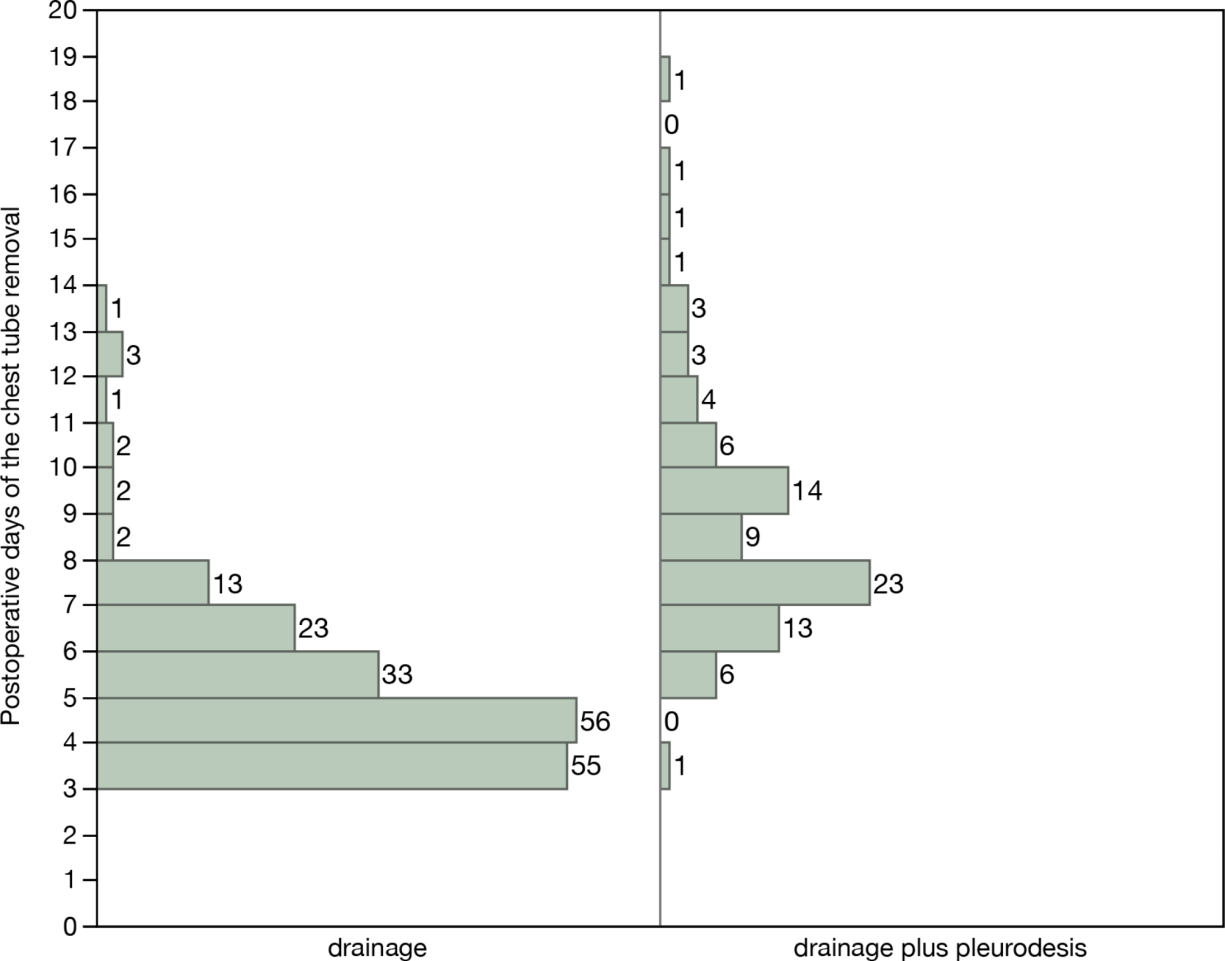
The number of patients per postoperative day of chest tube removal

Postoperative air leakage was observed in 277 patients. Among them, 191 were treated with drainage alone, while 86 underwent pleurodesis in addition to drainage. The characteristics of the patients who were treated with drainage and those of patients who were treated with drainage plus pleurodesis are shown in Table 1. There was no significant difference in patient background between the two groups. Figure 1 shows the postoperative days of chest tube removal in the two groups. The chest tube was removed after POD 7 in 24 (13%) patients in the drainage group (Fig. 1). Four patients prolonged air leaks more than 14 days. Although these patients required multiple pleurodesis, they did not have empyema or interstitial pneumonia.

**Table 1:** Characteristics of patients who were treated with drainage alone or drainage plus pleurodesis for postoperative air leaks

Variable	Drainage	Drainage plus pleurodesis	P-value
	n = 191	n = 86	
Age (years)	72 (66–76)	71 (67–77)	0.43
Male sex	139 (73%)	62 (72%)	0.91
Smoking history	142 (74%)	64 (74%)	0.99
Smoking Index	20 (0–30)	20 (0–20)	0.91
Emphysema	87 (46%)	36 (42%)	0.57
Interstitial pneumonia	48 (25%)	17 (20%)	0.43
FEV1/ FVC (%)	74.5 (68–79.5)	74 (67–81)	0.70
%VC (%)	96.7 (85.7–108.7)	97.5 (86.6–106.6)	0.68
%DLCO (%)	71.6 (55.2–83.7)	69.3 (57.2–86.4)	0.55
Surgical approach			0.025
Hybrid VATS	170 (89%)	68 (79%)	
RATS	9 (5%)	12 (14%)	
Open thoracotomy	12 (6%)	6 (7%)	
Surgical procedure			0.29
Lobectomy	116 (61%)	58 (67%)	
Segmentectomy	75 (39%)	28 (33%)	
Diagnosis			0.17
Adenocarcinoma	141 (74%)	70 (81%)	
Others	50 (26%)	16 (19%)	

Values are presented as number (%) or median (interquartile range). CT, computed tomography; DLCO, diffusion capacity of the lung for carbon monoxide; FEV1, forced expiratory volume in 1 s; FVC, forced vital capacity; VC, vital capacity

Detailed characteristics of the initial pleurodesis are presented in Table 2. Pleurodesis was performed a median of 5 (4–6) days after surgery. Talc was the most frequently used material for pleurodesis (in 52 [60%] patients), followed by autologous blood patches (in 20 [24%]), OK-432 (in 12 [14%]), minocycline (in one [1%]), and 50% glucose (in one [1%]). Fifty-two (60%) and 25 (29%) patients underwent pleurodesis once and twice, respectively (Table 2). Among the 34 patients who required multiple pleurodeses, two different materials were used for pleurodesis in 8 9%). The median number of days from initial pleurodesis to chest tube removal was 3 (2–5), and the median chest removal day was POD 8 (7–9). Two patients underwent a second surgery for air leak treatment: one received an autologous blood patch twice after POD 6, and the other received talc three times and OK-432 once after POD 3. One patient who were treated with OK432 developed a recurrent pneumothorax after drain removal. No grade 3 or more adverse events related to pleurodesis were observed. Four patients who underwent pleurodesis (Talc [n = 2], OK432 [n = 1], autologous blood patch [n = 1]) subsequently underwent second ipsilateral lung resection for secondary lung tumor. The patient who underwent autologous adhesion showed partial adhesions, whereas all those who underwent chemical pleurodesis showed subtotal adhesions.

**Table 2:** Detailed characteristics of pleurodesis

Variable	n = 86
The postoperative day of initial pleurodesis	5 (4–6)
Material used for initial pleurodesis	
Talc	52 (60%)
Autologous blood patch	20 (24%)
OK-432	12 (14%)
Others (Minocycline and 50% glucose)	2 (2%)
Frequency of pleurodesis performed	
1	52 (60%)
2	25 (29%)
≥3	9 (11%)
Days from initial pleurodesis to chest tube removal	3 (2–5)
Postoperative days of chest tube removal	8 (7–9)
Postoperative days of hospital stay	11 (9–14)

Values are presented as number (%) or median (interquartile range).

The differences in the characteristics of patients who received either of the three initial materials for pleurodesis are shown in Table 3. Interstitial pneumonia was the most frequently observed in the autologous blood patch group, followed by the talc group. No patient in the OK-432 group had emphysema or interstitial pneumonia. Diffusion capacity of the lung for carbon monoxide was lowest in the autologous blood patch group, followed by the talc and OK-432 groups. Although differences in patient characteristics among the three groups need to be considered, the efficacy of pleurodesis was nearly equivalent among the groups (Table 4). Approximately 90% of patients had their chest tube removed after up to two pleurodesis procedures (talc, 87%; autologous blood patch, 90%; OK-432, 92%; P = 0.56). There was no difference in the number of days from initial pleurodesis to chest tube removal among the materials used (talc, 3 (2–5) days; autologous blood patch, 3 (2–4) days; OK-432, 2 (1–6) days; P = 0.55). Multivariable logistic analysis showed that only more than 70 years was a risk factor of requiring two or more pleurodesis (odds ratio, 2.96: 95% confidence interval, 1.16–7.87: P. value, 0.029), but initial material was not (Table 5).

**Table 3:** Differences in the characteristics of patients who received either of the three initial materials for pleurodesis (n = 84)

Variable	Talc n = 52	Autologous blood patch n = 20	OK-432 n = 12	P-value
Age (years)	71 (67–78)	72 (65–77)	74 (67–78)	0.82
Male sex	40 (77%)	14 (70%)	7 (58%)	0.41
Smoking history	40 (77%)	17 (85%)	6 (50%)	0.08
Emphysema	21 (40%)	15 (75%)	0 (0%)	< 0.001
Interstitial pneumonia	5 (10%)	10 (50%)	0 (0%)	< 0.001
Pulmonary function				
FEV1/ FVC (%)	74 (67–81)	72.8 (62.9–80.1)	77.6 (70.4–84.0)	0.66
%VC (%)	95.4 (87.2–103.0)	99.1 (86–109.2)	101.7 (86–109.2)	0.74
%DLCO (%)	70 (54.3–84.4)	65.2 (54.8–77.3)	94.4 (72.8–102.6)	0.006
Surgical procedure				0.37
Lobectomy	34 (65%)	16 (80%)	7 (58%)	
Segmentectomy	18 (35%)	4 (20%)	5 (42%)	
Postoperative days of initial pleurodesis	5 (3–6)	5 (4–6)	4 (3–5)	0.11

Values are presented as number (%) or median (interquartile range). DLCO, diffusion capacity of the lung for carbon monoxide; FEV1, forced expiratory volume in 1 s; FVC, forced vital capacity; VC, vital capacity

**Table 4:** Outcomes based on the initial materials used for pleurodesis (n = 84)

	Talc n = 52	Autologous blood patch n = 20	OK-432 n = 12	P-value
Chest tube removal after initial pleurodesis	30 (58%)	11 (55%)	9 (75%)	0.49
Chest tube removal after up to twice pleurodesis	45 (87%)	18 (90%)	11 (92%)	0.56
Days from initial pleurodesis to chest tube removal	3 (2–5)	3 (2–4)	2 (1–6)	0.55
Postoperative days of chest tube removal	8 (7–10)	7 (7–11)	7 (6–8)	0.14
Postoperative length of hospital stay (days)	11 (9–16)	12 (9–14)	11 (8–13)	0.38

Values are presented as number (%) or median (interquartile range).

**Table 5:** Multivariable logistic analysis for risk of requiring two or more pleurodesis

Variable (reference)	Univariable OR (95% CI)	P-value	Multivariable OR (95% CI)	P-value
Age ≥ 70 years (< 70 years)	2.38 (0.93–6.08)	0.069	2.96 (1.16–7.87)	0.029
Emphysema yes (no)	1.57 (0.64–3.82)	0.320		
Interstitial pneumonia yes (no)	2.50 (0.74–8.45)	0.140	3.38 (0.95–12.01)	0.059
Segmentectomy (Lobectomy)	0.98 (0.39–2.48)	0.974		
Initial material				
Uni -talc	Ref			
Autologous blood patch	1.11 (0.39–3.15)	0.836		
OK-432	0.45 (0.11–1.88)	0.275		
Postoperative day at pleurodesis				
Within 4 days	Ref			
After 5 days	0.79 (0.33–1.90)	0.602		

CI, confidential interval; OR, odds ratio

We also compared the drainage time and hospital time between the early and standard groups. No differences in patients’ backgrounds, except for sex, were observed between the two groups (Table 6). Although the number of days from initial pleurodesis to chest tube removal were longer in the early group than that in the standard group (4 (3–6) days vs. 3 (2–4), P = 0.028), the drainage time and hospital stay were significantly shorter in the early group than those in the standard group (7 (6–8) days vs. 9 (7–10) days, P = 0.004; 10 (8–14) days vs. 11 (10–15) days; P = 0.048, respectively) (Table 7).

**Table 6:** Patients characteristics of the early and standard groups (n = 86)

Variable	Early group (pleurodesis within 4 days) n = 35	Standard group (pleurodesis on 5 days or later) n = 51	P-value
Age	71 (68–79)	71 (66–77)	0.79
Male sex	30 (86%)	32 (63%)	0.02
Smoking history	27 (77%)	37 (73%)	0.63
Emphysema	13 (37%)	23 (45%)	0.46
Interstitial pneumonia	5 (14%)	12 (23%)	0.29
FEV1/ FVC (%)	78 (70.2–83.8)	73.1 (64.2–79.9)	0.05
%VC (%)	100.5 (90.3–109.2)	97 (85.5–106.1)	0.27
DLCO (%)	77.7 (61.4–93.8)	68.1 (54–82.9)	0.15
Surgical procedure			0.45
Lobectomy	22 (63%)	36 (71%)	
Segmentectomy	13 (34%)	15 (29%)	
Material used			0.15
Talc	23 (66%)	29 (59%)	
Autologous blood patch	5 (14%)	15 (31%)	
OK-432	7 (20%)	5 (10%)	
Postoperative days of initial pleurodesis	3 (3–4)	5 (5–6)	< 0.001

Values are presented as number (%) or median (interquartile range). DLCO, diffusion capacity of the lung for carbon monoxide; FEV1, forced expiratory volume in 1 s; FVC, forced vital capacity; VC, vital capacity

**Table 7:** Pleurodesis outcomes of the early and standard groups (n = 86)

Variable	Early group (pleurodesis within 4 days) n = 35	Standard group (pleurodesis on 5 days or later) n = 51	P-value
Chest tube removal after initial pleurodesis	20 (57%)	32 (63%)	0.60
Chest tube removal after up to twice pleurodesis	29 (83%)	47 (92%)	0.19
Days from initial pleurodesis to chest tube removal	4 (3–6)	3 (2–4)	0.03
Postoperative days of chest tube removal	7 (6–8)	9 (7–10)	0.004
Chest tube removal within seven days	22 (63%)	21 (41%)	0.05
Postoperative length of hospital stay (days)	10 (8–14)	11 (10–15)	0.05

Values are presented as number (%) or median (interquartile range).

## Discussion

The present study revealed that early postoperative pleurodesis can shorten the drainage time and hospital stay of patients who had undergone lung resection. The timing of pleurodesis has not previously been discussed because it is generally performed for prolonged air leaks, which is defined as an air leak lasting for more than 5 days according to the Society of Thoracic Surgeons and the European Society of Thoracic Surgeons General Thoracic Surgery Database [12]. Therefore, pleurodesis is usually performed after POD 5 [4–6, 14, 15]. However, it is unclear whether pleurodesis after POD 5 is appropriate. The present study revealed the efficacy of early postoperative pleurodesis performed within 4 PODs. Reducing the duration of drainage provides several benefits, including decreased hospitalization costs, chest tube-related discomfort and pain, and morbidity [1, 2, 8]. In particular, prolonged air leaks (> 7 days) are associated with an increased risk for empyema and pneumonia [1]; therefore, sealing air leaks as early as possible is important. However, early postoperative pleurodesis is sometimes performed in patients who do not need it. Our study revealed that 24 (13%) patients who were treated with drainage had their chest tube removed after POD 7 (Fig. 1). This suggests that even if pleurodesis was delayed until after POD 5, some patients could have recovered with drainage alone. Several complications associated with pleurodesis have been reported. Talc and OK-432 administration induce acute respiratory distress syndrome or acute exacerbation of interstitial pneumonia [10, 11, 16]. In addition, the risk of chemical pneumonia due to the aspiration of pleurodesis material through the fistula should be noted [17]. Since we did not administer chemical materials for pleurodesis in patients with large air leaks or severe interstitial pneumonia, no lung injury or exacerbation of interstitial pneumonia was observed. Since autologous blood patches are reportedly safer than chemical pleurodesis [18, 17], we often used autologous blood patches in patients with interstitial pneumonia, which is a high-risk lung condition for chemicals. Thus, early postoperative pleurodesis could be safe when pleurodesis is performed based on patient status and the severity of air leakage. Two recent retrospective studies [19, 20] have reported that the long-term adverse effects of pleurodesis include decreased pulmonary function 12 months after surgery. However, due to their small sample size and because it was only observed at one institution, prospective trials are needed to confirm this.

The present study revealed that although the patients’ backgrounds differed among the autologous blood patch, talc, and OK-432 groups, the efficacies of the three materials would be considered comparable. There was no significant difference in the chest tube removal rate based on the number of pleurodesis performed or the days from initial pleurodesis to chest tube removal. The three materials have different mechanisms for sealing air leaks. Talc and OK-432 cause an intense intrapleural inflammatory response, leading to the sealing of the visceral pleura [21–23]. Two different mechanisms have been reported for autologous blood patches; one is that it induces an intrapleural inflammatory reaction leading to pleurodesis as well as chemical pleurodesis, and another is that coagulated blood directly seals the site of the air leak [24, 25]. Using different materials could increase the therapeutic effects of air leaks in patients who require pleurodesis more than three times. In our study, seven of eight patients who received two different materials recovered and had their chest tubes removed. The remaining patient underwent surgery for persistent air leaks after receiving talc and OK-432.

The present results should be interpreted in the light of some limitations. First, this was a retrospective study conducted at a single institution. Second, the amount of air leakage at the time pleurodesis was performed was unclear and might have differed between patients treated with pleurodesis and those treated with drainage. Therefore, we could not compare the efficacies of pleurodesis and drainage for postoperative air leaks. Third, there is no standardized protocol for pleurodesis or strategy for treating air leaks at our institution. The surgeons decide on the materials to use and the timing of pleurodesis based on the patient’s status or the amount of air leakage. Therefore, patient characteristics differed among the three materials, and it was difficult to establish accurate comparison groups. Finally, there was no data about the effects of pleurodesis on pulmonary function in this study. Since the mechanism of autologous blood patch is different from chemical pleurodesis, its effects on postoperative pulmonary function remain unknown. Randomized trials are required to accurately assess the ideal materials and timing for pleurodesis.

## Conclusions

Early postoperative pleurodesis within 4 days of surgery can be an option to shorten the drainage time and length of hospital stay. The efficacies of autologous blood patches, talc, and OK-432 would be considered comparable, although patient backgrounds differed among the three groups. Randomized controlled trials are required to confirm these findings.

## Data Availability

The datasets used and analysed during the current study are available from the corresponding author on reasonable request.

## Abbreviations

POD: postoperative day
